# Causal relationship of gut microbiota with diabetic nephropathy: a Mendelian randomization analysis

**DOI:** 10.3389/fmicb.2023.1281361

**Published:** 2024-01-03

**Authors:** Wei Yan, Ying Ge, Lina Wang, Yuntao Wang, Daikun He

**Affiliations:** ^1^Department of General Practice, Jinshan Hospital, Fudan University, Shanghai, China; ^2^Department of General Practice, Zhongshan Hospital, Fudan University, Shanghai, China; ^3^Center of Emergency and Critical Care Medicine, Jinshan Hospital, Fudan University, Shanghai, China

**Keywords:** diabetic nephropathy, gut microbiota, Mendelian randomization analysis, causal relationship, kidney damage

## Abstract

**Background:**

Patients with DN (diabetic nephropathy) show remarkable variations in their gut microbiota composition. However, to date, no study has shown whether a causal relationship exists between gut microbiota composition and DN.

**Methods:**

Here, we performed a two-sample Mendelian randomization (MR) investigation for identifying causal associations of gut microbiota with DN. Gut microbiota genetic data were gathered from the recent genome-wide association study pooled data of the MiBioGen consortium, which included 24 cohorts and 18,340 individuals.

**Results:**

IVW(Inverse variance weighting) revealed that *Verrucomicrobia* [odds ratio (OR) = 1.390; 95% confidence interval (CI) = 1.10–1.75; *p* = 0.005], *Peptostreptococcaceae* (OR = 1.284; 95% CI = 1.03–1.59; *p* = 0.012), *Verrucomicrobiaceae* (OR = 1.390; 95% CI = 1.10–1.75; *p* = 0.005), *Akkermansia* (OR = 1.390; 95% CI = 1.10–1.75; p = 0.005), *Butyricimonas* (OR = 1.261; 95% CI = 1.02–1.55; *p* = 0.031), *Catenibacterium* (OR = 1.278; 95% CI = 1.02–1.59; *p* = 0.030).

**Conclusion:**

Two-sample MR analysis identified 12 microbial taxa in gut microbiota (one of which is yet to be officially named) that showed significant causal associations with DN; 8 of these taxa significantly increased the risk of DN, while the remaining 4 taxa (including the one without an official name) reduced the risk of DN. The precise mechanisms influencing the interactions of gut microbiota with DN occurrence remain unclear; hence, additional investigations should be conducted to clarify these mechanisms.

## Introduction

Diabetic nephropathy (DN), a frequently occurring diabetic complication, leads to high mortality in patients with diabetes (Alicic et al., 2017). DN is mainly characterized by glomerulosclerosis caused by microangiopathy due to poor blood glucose control over a long term. DN shows clinical manifestations as proteinuria, hypertension, and incremental decline in renal function (Oshima et al., 2021). Currently, the four main areas of treatment for DN include blood pressure control, glycemic control, reducing cardiovascular disease risk, and suppressing the renin-angiotensin-aldosterone system (Yang et al., 2021). Because DN presents very limited treatment scope currently, it is essential to constantly explore and discover new therapeutic targets.

Recent studies have described many risk factors for DN (Alicic et al., 2017), among which, hyperglycemia and hypertension are the main risk factors (Samsu, 2021). A close link between gut microbiota and DN has been noted. Patients with DN show remarkable differences in their gut microbiota composition; moreover, gut microbiota could influence DN occurrence and development via the gut-kidney axis. Therefore, gut microbiota disruption is considered a risk factor for DN occurrence (Burgess et al., 2019; Cai et al., 2020; Wu et al., 2020; Iatcu et al., 2021; Cai et al., 2022; Deng et al., 2022; Guo et al., 2022; Luo et al., 2022; Lv et al., 2022; Nagase et al., 2022; Ni et al., 2022; Zhang et al., 2022). However, these observational studies did not show a causal relationship of gut microbiota with DN.

Mendelian randomization (MR) is used as an inference analysis for determining causal relationships. It functions on the principle of Mendelian inheritance, and it infers the causal relationship of exposure factors with outcomes through instrumental variables (IVs) as single nucleotide polymorphisms (SNPs) or genetic variants (Davey Smith and Hemani, 2014). MR analysis can determine the underlying biological mechanisms, avoid confounding factor interference, and ensure causal inference accuracy (Birney, 2022). Here, we implemented the MR approach for investigating the causal relationship of gut microbiota composition with DN. We noted that various gut bacterial taxa show a causal relationship with DN.

## Materials and methods

### Study design

The exposure factor was 211 bacterial taxa of gut microbiota, and the outcome was DN. We preliminarily screened gut microbiota significantly associated with DN. The detailed MR analysis was performed with the following assumptions: (1) an association between IVs and exposure factors, (2) no relationship between IVs and confounding factors, and (3) influence of IVs on outcomes only through exposure factors. F-statistic of SNPs was calculated to investigate bias existence due to a weak IV (Burgess et al., 2011).

### Data sources

Gut microbiota genetic data were collected using the pooled data of the recent genome-wide association study (GWAS) from the MiBioGen consortium, which included 24 cohorts and 18,340 individuals (URL for data download: https://mibiogen.gcc.rug.nl/). Gut microbiota composition was analyzed according to three variable regions (V1–V2, V3–V4, and V4) of the 16S rRNA gene. Microbiota quantitative trait locus (mbQTL) mapping was applied for identifying genetic variations affecting the relative abundance of microbial taxa (Kurilshikov et al., 2021).

The FinnGen Biobank, which includes data from 213,746 European individuals (210,463 controls and 3,283 cases), was used to retrieve genetic data on diabetic kidney disease. The Integrative Epidemiology Unit OpenGWAS database was searched using “diabetic nephropathy” as the keyword, and two related GWASs were retrieved. Of these, one GWAS [GWAS ID: Finn-b-DM_NEPHROPATHY (more information available at https://gwas.mrcieu.ac.uk)] was selected as it was relatively recent and had a large number of SNPs, with the phenotype as “Diabetic nephropathy.”

### Statement of ethics

The used pooled GWAS data are publicly available. Informed consent as well as institutional ethical approval were received for the original trial. Hence, additional ethical approval was waived off for this study.

### IV selection

Six taxonomic levels were used to classify the 211 bacterial taxa. Among these levels, the genus was the most specific and smallest taxonomic level. IVs were selected according to four criteria: (1) SNPs linked with the gut bacterial taxa were chosen as potential IVs according to *p* < 5.0 × 10^−5^ as the significance threshold; (2) to detect independent SNPs, the European genotype of 1,000 genomes was used as the reference genome, and the clumping threshold of linkage disequilibrium was r^2^ < 0.001, with 10,000 kb as clumping window size; (3) palindromic SNPs were deleted from the curated data; and (4) MR-PRESSO (Mendelian Randomization Pleiotropy RESidual Sum and Outlier) as well as MR-Egger regression were applied for detecting potential pleiotropy and for removing pleiotropy effect by excluding outliers (Bowden et al., 2019). To evaluate the strength with each SNP shows an association with the exposure factor, the F-statistic was determined for each bacterial taxon with the below-mentioned formula, and the IVs’ strength was estimated with the *F*-statistic.


$$F=\frac{R^{2}\left(n-1-k\right)}{\left(1-R^{2}\right)k}$$

*F*-statistic values of ≥10 and < 10 indicated no bias of Ivs (Bowden et al., 2019) and a weak IV that should be eliminated, respectively.

### Statistical analysis

Statistical analysis software used for the data analysis included MR-Presso version 1.0, RStudio version 4.2.1, and TwoSampleMR version 0.5.6. Inverse variance weighting (IVW) was applied as the main statistical test. Weighted median, MR-Egger regression, MR-PRESSO, and simple mode as well as weighted mode methods were additionally used. IVW was chosen as the preferred statistical method because of its higher detection efficiency than the other four MR methods. We selected these statistical tests based on the following logical reasons: (1) IVW is a weighted linear regression model that is mainly used in Mendelian studies of multiple IVs. It assumes that genetic variants are relevant IVs and has strong detection power for causal relationships (Bowden et al., 2017); (2) MR-Egger regression summarizes data and assumes genetic variants to have horizontal gene pleiotropy; in this method, the pleiotropy of IVs is estimated through the intercept generated by weighted linear regression while considering the existence of the intercept (Burgess and Thompson, 2017a). MR-Egger considers some degree of pleiotropy of the IVs; this implies retention of the outcome effect at the exposure effect value of zero (intercept). The MR-Egger intercept term in the MR-Egger intercept test is compared to zero, with a larger variation indicating greater horizontal pleiotropy (Burgess and Thompson, 2017b); (3) MR-PRESSO adds each SNP’s residuals to estimate the magnitude of horizontal pleiotropy, and after adjusting for horizontal pleiotropy, IVW results are derived. The MR-PRESSO global test evaluates IVs’ overall pleiotropy level, while the MR-PRESSO outlier test evaluates aberrant SNPs responsible for the overall pleiotropy level; and (4) the weighted median method adjusts for ineffective IVs’ effects and produces robust estimates even in the presence of 50% of ineffective IVs. We performed leave-one-out analyses (in which the impact of remaining SNPs is calculated after excluding one SNP at a time), which assess how an outlier affects the outcomes. Furthermore, causality direction was determined with a reverse MR analysis.

## Results

### IV selection and outcomes of initial MR analysis

We initially chose 2,561 SNPs as IVs (all IVs) for the 211 taxa, and finally, 12 taxa were isolated according to *p* < 0.05 by IVW. Considering the influence of confounding factors, we used PhenoScanner to query the SNPs with the abovementioned positive results. No SNPs were related to the confounding factors.

### Detailed MR results

**Figure 1:**
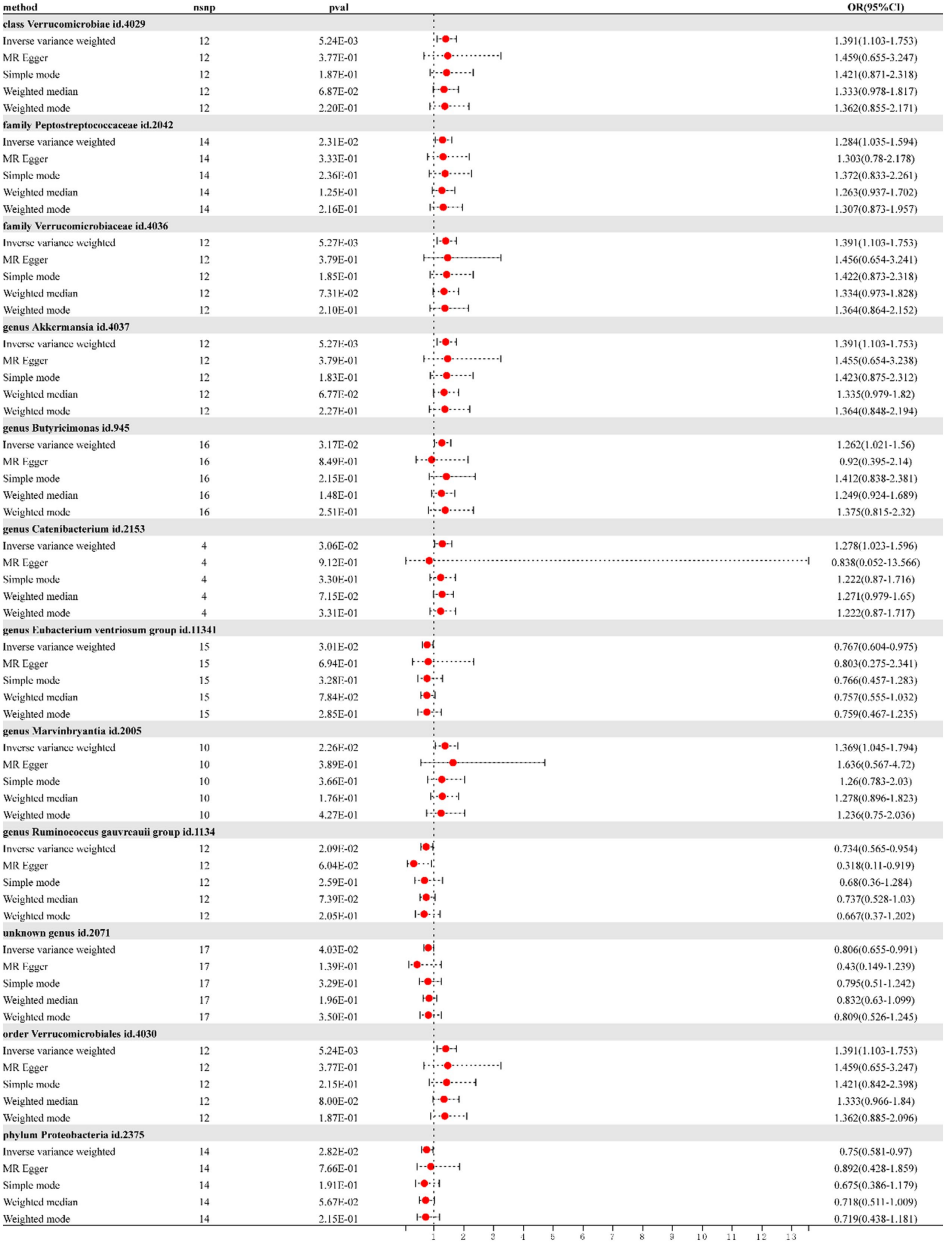
Forest plot of the effect of gut microbiota on diabetic nephropathy; OR, odds ratio; CI, confidence interval.

According to IVW, the presence of class *Verrucomicrobia* [odds ratio (OR) = 1.390; 95% confidence interval (CI) = 1.10–1.75; *p* = 0.005], family *Peptostreptococcaceae* (OR = 1.284; 95% CI = 1.03–1.59; *p* = 0.012), family *Verrucomicrobiaceae* (OR = 1.390; 95% CI = 1.10–1.75; p = 0.005), genus *Akkermansia* (OR = 1.390; 95% CI = 1.10–1.75; p = 0.005), genus *Butyricimonas* (OR = 1.261; 95% CI = 1.02–1.55; *p* = 0.031), genus *Catenibacterium* (OR = 1.278; 95% CI = 1.02–1.59; *p* = 0.030), genus *Marvinbryantia* (OR = 1.369; 95% CI = 1.04–1.79; *p* = 0.022), and order *Verrucomicrobiales* (OR = 1.390; 95% CI = 1.10–1.75; p = 0.005) in gut microbiota increased risk for DN. However, genus *Eubacterium ventriosum* group (OR = 0.767; 95% CI = 0.60–0.97; p = 0.030), genus *Ruminococcus gauvreauii* group (OR = 0.734; 95% CI = 0.56–0.95; *p* = 0.020), phylum *Proteobacteria* (OR = 0.750; 95% CI = 0.58–0.97; *p* = 0.028), and the unknown genus ID 2071 (currently unnamed; OR = 0.805; 95% CI = 0.65–0.99; *p* = 0.040) in gut microbiota reduced DN risk. As shown in the forest plot (Figure 1).

As shown in the scatterplot, except *Butyricimonas* and *Catenibacterium*, the remaining gut microbiota were all in the same direction. Because the IVW method has the strongest statistical power, our results predominantly relied on the IVW method. Horizontal pleiotropy was estimated primarily with MR-Egger. The MR-PRESSO global and MR-Egger intercept tests exhibited a high likelihood of horizontal pleiotropy (sensitivity analysis; *p* > 0.05); moreover, in heterogeneity test, all I^2^ values were < 50%. However, because *p* value was >0.05, the results were unaffected by bias due to heterogeneity (see Table 1).

**Table 1:** Significant MR analysis results in the discovery samples.

Bacterial taxa (exposure)	MR method	No. of SNP	*F*-statistic	OR	95% CI	*p*-value
Class *Verrucomicrobia*	IVW	12	418.69	1.390	1.10–1.75	0.005
	MR-Egger	12		1.458	0.65–3.24	0.376
	SM	12		1.420	0.87–2.31	0.186
	WME	12		1.333	0.97–1.81	0.068
	WM	12		1.362	0.85–2.17	0.220
Family *peptostreptococcaceae*	IVW	14	506.76	1.284	1.03–1.59	0.023
	MR-Egger	14		1.302	0.77–2.17	0.332
	SM	14		1.372	0.83–2.26	0.236
	WME	14		1.263	0.93–1.70	0.124
	WM	14		1.307	0.87–1.95	0.215
Family *Verrucomicrobiaceae*	IVW	12	449.89	1.390	1.10–1.75	0.005
	MR-Egger	12		1.456	0.65–3.24	0.378
	SM	12		1.422	0.87–2.31	0.184
	WME	12		1.333	0.97–1.82	0.073
	WM	12		1.363	0.86–2.15	0.209
Genu *Akkermansia*	IVW	12	450.34	1.390	1.10–1.75	0.005
	MR-Egger	12		1.455	0.65–3.23	0.379
	SM	12		1.422	0.87–2.31	0.182
	WME	12		1.335	0.97–1.82	0.067
	WM	12		1.363	0.84–2.19	0.227
Genu *Butyricimonas*	IVW	16	644.69	1.261	1.02–1.55	0.031
	MR-Egger	16		0.919	0.39–2.14	0.849
	SM	16		1.412	0.83–2.38	0.214
	WME	16		1.249	0.92–1.68	0.148
	WM	16		1.375	0.81–2.32	0.251
Genu *Catenibacterium*	IVW	4	485.29	1.278	1.02–1.59	0.030
	MR-Egger	4		0.837	0.05–1.35	0.912
	SM	4		1.222	0.87–1.71	0.330
	WME	4		1.271	0.97–1.65	0.071
	WM	4		1.222	0.86–1.71	0.331
Genu *Eubacterium ventriosum*_group	IVW	15	419.38	0.767	0.60–0.97	0.030
	MR-Egger	15		0.802	0.27–2.34	0.693
	SM	15		0.765	0.45–1.28	0.327
	WME	15		0.756	0.55–1.03	0.078
	WM	15		0.758	0.46–1.23	0.285
Genu *Marvinbryantia*	IVW	10	351.21	1.369	1.04–1.79	0.022
	MR-Egger	10		1.635	0.56–4.71	0.389
	SM	10		1.260	0.78–2.02	0.366
	WME	10		1.277	0.89–1.82	0.176
	WM	10		1.235	0.75–2.03	0.427
Genu *Ruminococcus gauvreauii* group	IVW	12	367.75	0.734	0.56–0.95	0.020
	MR-Egger	12		0.318	0.11–0.91	0.060
	SM	12		0.679	0.35–1.28	0.259
	WME	12		0.737	0.53–1.02	0.074
	WM	12		0.666	0.37–1.20	0.205
unkown Genus id 2071	IVW	17	573.71	0.805	0.65–0.99	0.040
	MR-Egger	17		0.430	0.14–1.23	0.139
	SM	17		0.795	0.50–1.24	0.329
	WME	17		0.832	0.63–1.09	0.198
	WM	17		0.809	0.52–1.24	0.355
Order *Verrucomicrobiales*	IVW	12	450.08	1.390	1.10–1.75	0.005
	MR-Egger	12		1.459	0.65–3.25	0.376
	SM	12		1.421	0.84–2.39	0.215
	WME	12		1.333	0.97–1.84	0.079
	WM	12		1.362	0.88–2.09	0.187
Phylum *Proteobacteria*	IVW	14	325.31	0.750	0.58–0.97	0.028
	MR-Egger	14		0.892	0.43–1.86	0.766
	SM	14		0.674	0.38–1.18	0.190
	WME	14		0.718	0.51–1.01	0.056
	WM	14		0.719	0.44–1.18	0.215

MR, Mendelian randomization; No. of SNP is the number of SNPs being used as IVs; IVW, Inverse variance weighted; SM, Simple mode; WME, Weighted median Estimator; WM, Weighted mode; OR, odds ratio; CI, confidence interval.

**Figure 2:**
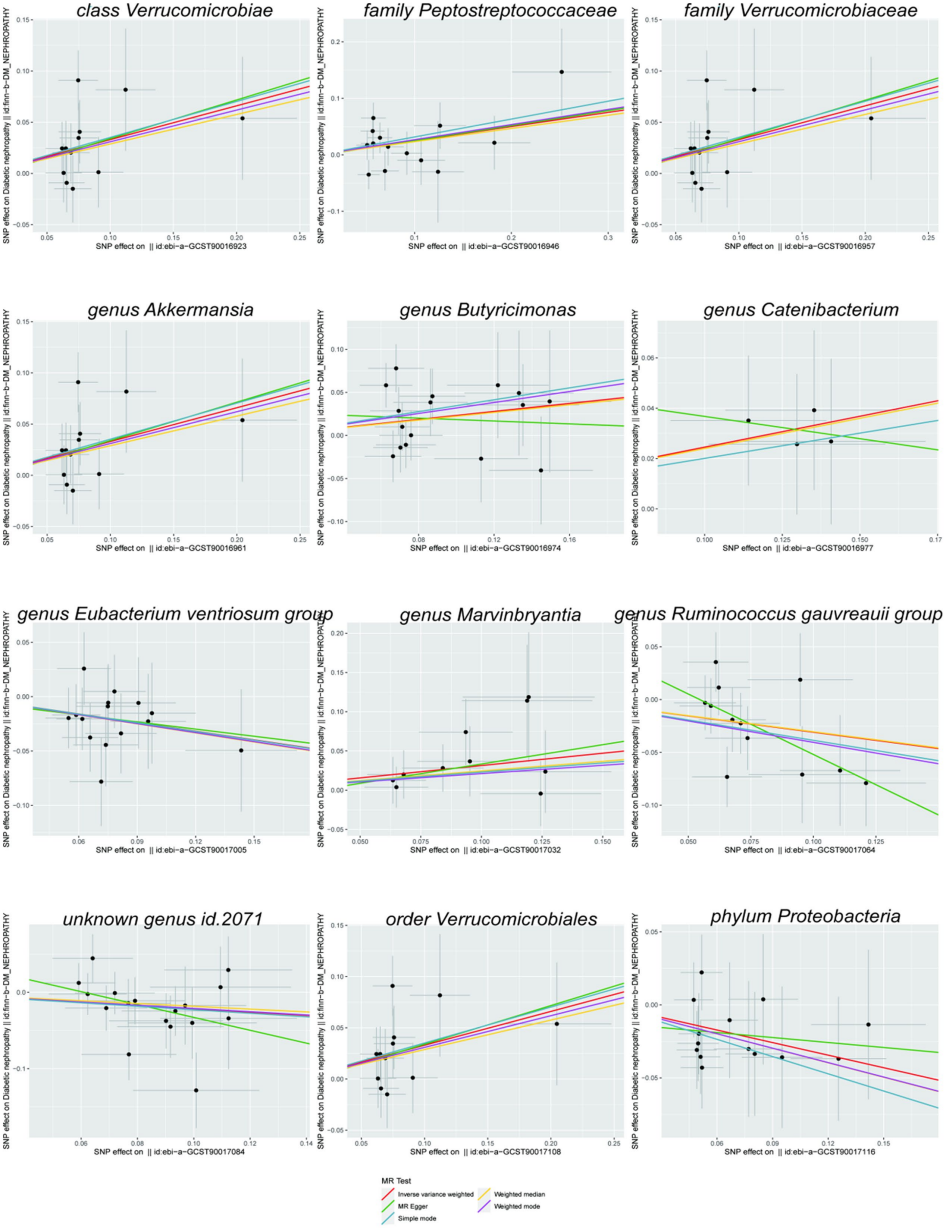
Scatterplots of the effect of gut microbiota on diabetic nephropathy.

**Figure 3:**
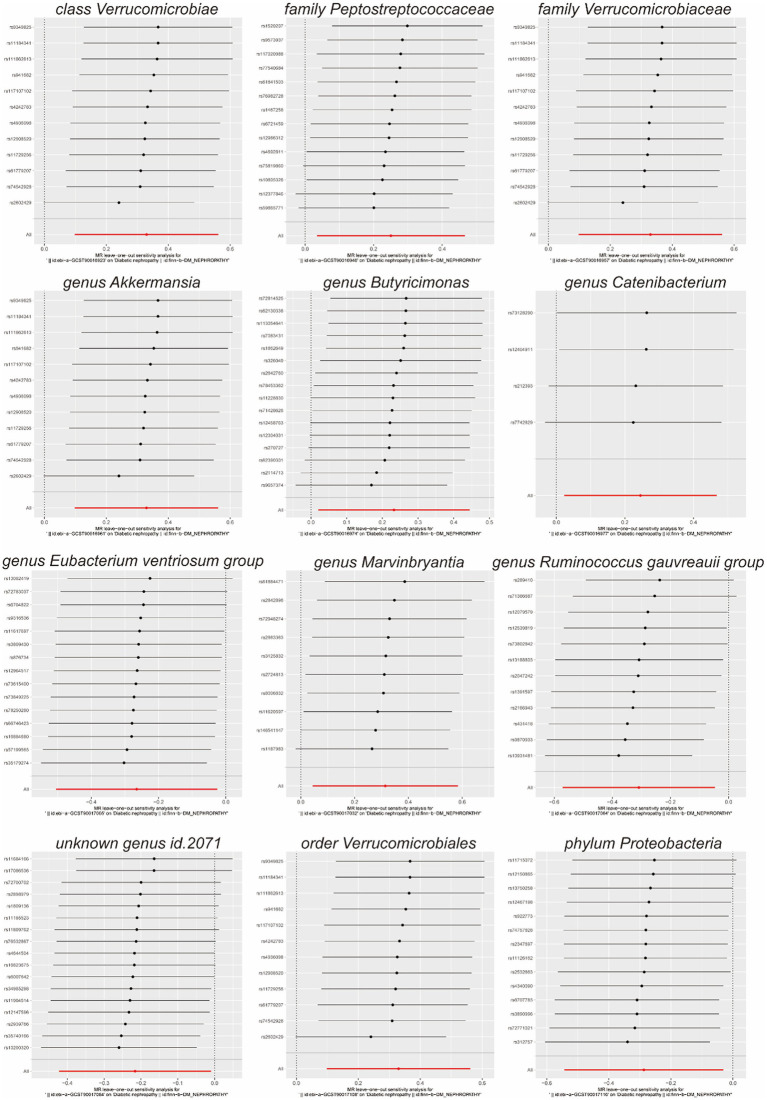
Leave-one-out plots of the effect of gut microbiota on diabetic nephropathy.

**Figure 4:**
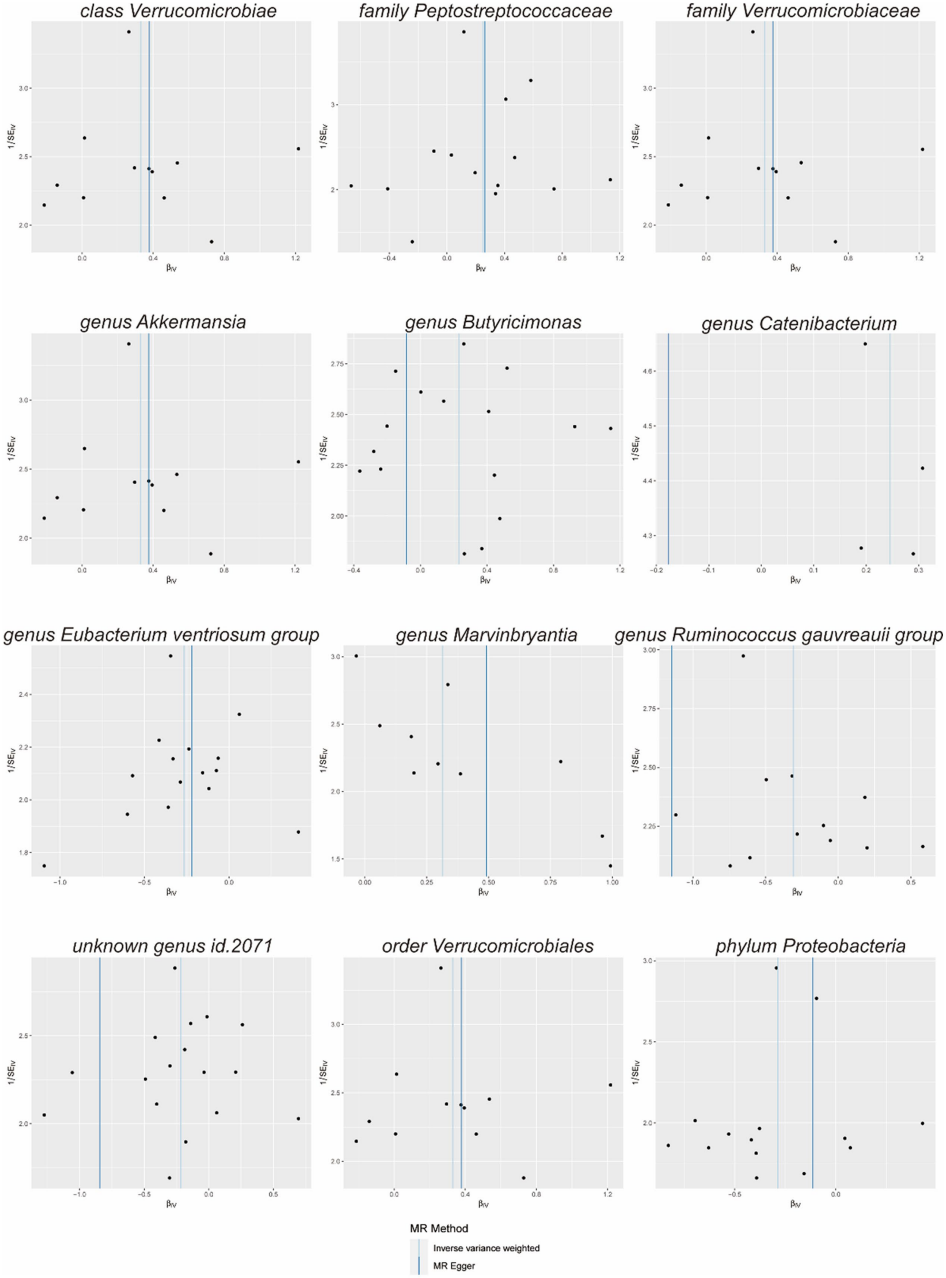
Funnel plot of the effect of gut microbiota on diabetic nephropathy.

Scatterplots (Figure 2) and leave-one-out plots (Figure 3) revealed *Verrucomicrobia*, *Verrucomicrobiaceae*, *Akkermansia*, and *Verrucomicrobiales* as outliers. No outliers were noted in the MR-PRESSO outlier test. The leave-one-out plots clarified that none of the SNPs largely influenced the effect estimate; this finding indicated that the causality was relatively stable. The funnel plot (Figure 4) showed that the distribution of each IV was symmetric and unbiased.

## Discussion

By conducting a two-sample MR analysis, we evaluated the causal relationship of gut microbiota composition with risk for DN. We observed that *Verrucomicrobia, Peptostreptococcaceae*, *Verrucomicrobiaceae*, *Akkermansia*, *Butyricimonas*, *Catenibacterium*, *Marvinbryantia*, and *Verrucomicrobiales* increased the risk of diabetic kidney disease, while *E. ventriosum* group, *R. gauvreauii* group, *Proteobacteria*, and unknown genus ID 2071 exhibited a protective effect against DN. This suggests that the gut microbiota significantly influences the development and occurrence of DN.

Interestingly, this study revealed that *Akkermansia* increased the risk of DN. Numerous studies have demonstrated that *Akkermansia* is a crucial intestinal probiotic. The lack or decreased abundance *Akkermansia* was associated with obesity, diabetes, hepatic steatosis, inflammation and tumors (Cani et al., 2022). The mechanism affecting these metabolic diseases was the short-chain fatty acids (SCFAs) produced by *Akkermansia* bind to GPR43 and PR41, thereby stimulating GLP1 and GLP2 secretion and mucus production and secretion, consequently regulating the glucose metabolism and intestinal function (Cani et al., 2022). In animal experiments, *Akkermansia* stimulates host cells to produce specific bioactive lipids, stimulate GLP1 and GLP2 secretion, and activates inflammatory pathway and fatty acid oxidation (Cekanaviciute et al., 2017); However, a recent human study revealed that the gut microbiota of patients with chronic kidney disease (CKD) exhibited significantly high levels of five bacterial genera, including *Akkermansia*, indicating that *Akkermansia* is an important pathogen associated with CKD progression and may be the key gut microbe causing CKD progression. Other studies have reported that the abundance of *Akkermansia* is positively correlated with SCr and BUN levels and negatively correlated with eGFR and hemoglobin levels (Ren et al., 2020). which suggests that *Akkermansia* plays a crucial role in the progression of CKD. Production of uremic toxins may be the mechanism by which *Akkermansia* affects renal function, which concurs with our findings. *Akkermansia* is highly associated with metabolic disease significantly as it reduces the risk of diabetes. However, our research showed that *Akkermansia* increased the risk of DN. We deduced that *Akkermansia* could reduce the risk of diabetes before the development of diabetes, and if diabetes is already developed, *Akkermansia* could increase the risk of DN by affecting renal function. However, more clinical studies are needed to confirm this. Our results show that *Verrucomicrobia*, *Verrucomicrobiacea*e, and *Verrucomicrobiales* increased the risk of DN; however, *Verrucomicrobia*, *Verrucomicrobiaceae*, and *Verrucomicrobiales* are in the same bacterial group as *Akkermansia* and exhibit similar biological functions, suggesting that they probably increase the risk of DN by the same mechanism as *Akkermansia*. However, further studies are needed to confirm the specific mechanism.

Our study also discovered that *Peptostreptococcaceae*, *Butyricimonas*, *Catenibacterium,* and *Marvinbryantia* increased the risk of DN. Previous studies have shown that *Peptostreptococcacea*e and *Marvinbryantia* aggravate insulin resistance and are risk factors for type 2 diabetes (Chen et al., 2021). Moreover, it has been speculated that *Peptostreptococcaceae* and *Marvinbryantia* increase the risk of DN by increasing insulin resistance. The mechanism behind increasing insulin resistance may be that the SCFAs produced by *Peptostreptococcaceae* and *Marvinbryantia* affect glucose metabolism. *Butyricimonas* and *Catenibacterium* are anaerobic bacteria, and no related studies have been reported; However, it has been speculated that the increased risk of DN may be due to the production of the metabolite trimethylamine (TMA) (Ren et al., 2022); However, further studies are needed to confirm the exact mechanism.

Our study also showed that the *E. ventriosum* group, *R. gauvreauii* group, *Proteobacteria,* and unknown genus ID 2071 reduced the risk of DN. Other studies have shown that the *R. gauvreauii* group reduces insulin resistance (Chen et al., 2021), and it has been speculated that the *R. gauvreauii* group reduces the risk of DN by reducing insulin resistance. *E. ventriosum* group are important gut microbes in the healthy population. Although there has been no concrete report regarding this so far, it has been speculated that this group of microbes produces butyrate hydrochloride, thereby reducing insulin resistance. Additionally, it has been speculated that *Proteobacteria* may inhibit the growth of other harmful bacteria, reducing the risk of DN (Iatcu et al., 2021); However, the specific mechanism needs to be studied further. Furthermore, the unknown genus ID 2071 is currently unnamed and requires further investigation.

DN stands out as the most prevalent form of chronic kidney disease, primarily marked by damage to kidney function. In clinical practice, several disease, including rare genetic diseases like Fabry disease, Alport syndrome, and Bartter syndrome, can also result in kidney function damage. Fabry disease, an X-linked genetic disorder, arises mainly from mutations in the GLA gene on the chromosome, presenting with symptoms like proteinuria, reduced glomerular filtration rate, and hematuria. Diagnosis hinges on factors like family history, clinical presentation, laboratory tests, histopathological examination, and genetic testing (Chan and Adam, 2018). Alport syndrome exhibits key clinical features such as hematuria, proteinuria, and a progressive decline in kidney function, attributed to genetic mutations in the COL4A3, COL4A4, and COL4A5 genes (Kashtan, 2021). Bartter syndrome, a rare genetic tubulopathy, lacks a standardized diagnostic criterion. Clinical manifestations vary and often lack specificity, with hypokalemia and metabolic alkalosis being fundamental (Mrad et al., 2021). The diagnosis of these rare genetic diseases mainly relies on genetic testing.

The treatment landscape for DN covers a variety of medications, with sodium-glucose cotransporter 2(SGLT-2) inhibitors being prevalent. SGLT-2 inhibitors, a relatively recent class of antidiabetic drugs, have gained widespread clinical use due to their effective glucose-lowering properties. Acting primarily on renal tubules, SGLT-2 inhibitors facilitate the reabsorption of glucose in the primary urine. Extensive studies have indicated the renal protective effects of SGLT-2 inhibitors, although the exact mechanism remains unclear (Fitchett et al., 2019). The findings of this study offer new insights and potential targets for the treatment of DN.

Gut microbiota is an intricate microbial ecosystem existing mainly in the gastrointestinal tract of humans; its composition varies among individuals. It is a key component of gastrointestinal mucosal permeability and regulates the absorption and fermentation of dietary polysaccharides as well as lipid accumulation (Wu et al., 2023). Different microbial communities present in gut microbiota produce different metabolites, including choline, bile acids, neurotransmitters, short-chain fatty acids, small molecules, toxic substances, and inflammatory factors (Schoeler and Caesar, 2019). Based on metabolite production, gut microbiota participates in several physiological processes in various organs, such as signal transduction and energy metabolism (Nishida et al., 2018). These interacting pathways between the gut and the organ, termed the “gut-organ axis,” for example, the axis of gut-kidney, gut-liver, gut-bone, and gut-brain (Ahlawat and Asha, 2021), are highly important in sustaining the functions of several organs. Among these interacting pathways, gut microbiota significantly affects the gut-kidney axis. Intestinal flora affects renal function by synthesizing SCFAs, p-cresyl sulfate, trimethylamine N-oxide (TMAO), indoxyl sulfate, and other compounds (Zhang et al., 2021). SCFAs are essential for the integrity of intestinal epithelial cells and energy balance. They may alleviate hypoxic damage to renal epithelial cells by promoting mitochondrial biogenesis. Indoxyl sulfate and p-cresyl sulfate bind to blood albumin and are secreted by renal tubules. If uremic residual solutes accumulate in the body, they can accelerate glomerulosclerosis and kidney disease progression (Vanholder et al., 2014). As shown by previous studies, TMAO concentrations are 20-fold higher in end-stage renal disease patients than in healthy controls (Zeng et al., 2021). Elevated TMAO levels can lead to renal tubulointerstitial fibrosis and participate in the pathophysiological process of atherosclerosis.

## Conclusion

In summary, a two-sample MR analysis revealed 12 taxa of gut microbiota (including one with no official name) to be causally associated with DN. The specific mechanisms responsible for this association, however, remain unclear. Elucidation of these mechanisms responsible for gut microbiota effect on DN will require additional studies.

## Data availability statement

The datasets presented in this study can be found in online repositories. The names of the repository/repositories and accession number(s) can be found in the article/Supplementary material.

## Ethics statement

Ethical approval was not required for the study involving humans in accordance with the local legislation and institutional requirements. Written informed consent to participate in this study was not required from the participants or the participants' legal guardians/next of kin in accordance with the national legislation and the institutional requirements.

## Author contributions

WY: Data curation, Formal analysis, Funding acquisition, Methodology, Software, Writing – original draft. YG: Software, Writing – original draft. LW: Software, Writing – review & editing. YW: Software, Writing – review & editing. DH: Writing – review & editing.
